# Methanogenic activities signatures in broilers fed black soldier fly (*Hermetia illucens*) larvae-based diets

**DOI:** 10.1016/j.bbrep.2026.102441

**Published:** 2026-01-08

**Authors:** Deborah Oluwaferanmi Ibiwoye, Opeyemi Adetola Oladejo, Oluwatomisin Aderonke Akinsola, Aruna Olasekan Adekiya, Olufemi Mobolaji Alabi, Ayantade Dayo Ayansina, Samuel Olatunde Dahunsi

**Affiliations:** a Microbiology Programme, College of Agriculture, Engineering and Science, Bowen University, Iwo, Nigeria; b Department of Biological Sciences, Joseph Ayo Babalola University, Ikeji-Arakeji, Osun State, Nigeria; c Agriculture Programme, College of Agriculture, Engineering and Science, Bowen University, Iwo, Nigeria; d The Radcliffe Institute for Advanced Study, Harvard University, Cambridge, MA, United States of America

**Keywords:** Animal production and health, Black soldier fly, Cleaner production, Circular bioeconomy, Food security, Methanogenesis

## Abstract

Methanogenic archaea in the avian gut contribute to hydrogen turnover and thus play a role in fermentative efficiency and greenhouse gas emission. While black soldier fly larvae meal (BSFLM) has been explored as a replacement for fishmeal in broiler diets, less is known about how varying BSFLM inclusion levels affect methanogenesis pathways and enzyme activities in the gut microbial community. This study investigated the presence, relative abundance, and shifts in methanogenesis enzyme pathways in broilers fed diets with increasing levels of BSFLM. Arbor Acre Plus broiler chicks were allocated to five dietary treatments over 8 weeks. Diets replaced fishmeal with BSFLM at 0 % (control), 25 %, 50 %, 75 %, and 100 % levels. Cecal samples were collected post-mortem, DNA extracted, and the bacterial 16S rRNA (V1–V9) region sequenced on PacBio Sequel IIe. Functional predictions via PICRUSt2 were used to identify KEGG orthologs associated with methanogenesis (e.g. K00200–K00205 for FMD, K00399 etc. for MCR, methyltransferases, etc.). Enzyme detection across treatments was assessed qualitatively and semi-quantitatively (e.g. “low”, “moderate”, “strong”) based on relative abundance. Key methanogenic enzymes (including FMD, MCH, MTD, F420-dependent enzymes, methyltransferases, and MCR) were profiled, and their activity compared across treatments. Correlations were examined and predicted functional capacity via PICRUSt2. Methanogenesis-related pathways were detectable in all dietary treatments. In the control (0 % BSFLM), enzyme levels were minimal, reflecting low background methanogenic potential. At 25 % BSFLM inclusion (T2), there was strong detection of hydrogenotrophic pathway enzymes (FMD, coenzyme F420 hydrogenase, MCR) and moderate presence of methylotrophic methyltransferases, suggesting dominance of hydrogenotrophic methanogenesis. In the 50 % BSFLM treatment (T3), enzyme levels declined somewhat: hydrogenotrophic enzyme activity was moderate, and methylotrophic components were weaker. With 100 % BSFLM (T5), methylotrophic pathway enzymes (methanol- and methylamine-corrinoid protein Co-methyltransferases) were strongly detected, while hydrogenotrophic enzymes persisted but at low levels. These shifts have implications for gut fermentation efficiency, substrate (hydrogen and methyl compound) availability, nitrogen metabolism, and possibly GHG emission. Optimization of BSFLM inclusion levels may help balance production performance and environmental sustainability.

## Highlights

• Methanogenic archaea were detected in the caeca of broilers fed BSFLM.
• The PICRUSt2 tool was used to identify KEGG orthologs associated with methanogenesis.
• Key methanogenic enzymes (including FMD, MCH, MTD, F420-dependent enzymes, methyltransferases, and MCR) were profiled.
• Methanogenesis-related pathways were detectable in all dietary treatments with variations.
• These shifts have implications for gut fermentation efficiency, substrate availability, nitrogen metabolism, and GHG emission.

## 1 Introduction

The global poultry sector is under increasing pressure to find feed ingredients that reduce environmental impact while sustaining growth and gut health/wellbeing [1]. The most acceptable conventional protein sources have been soybean meal and fishmeal, but they have been under serious criticism owing to increasing cost, land use, deforestation, and gross instability in the supply chain. To remedy this situation therefore, insect-derived meals, in particular black soldier fly (*Hermetia illucens*) larvae meal (BSFLM), has emerged as a major attraction judging by their favorable nutrient profiles, high efficiency for waste and biomass conversion, and huge potential to partially or totally replace conventional feeds [[2], [3], [4]].

Meanwhile, the gut microbiome of poultry birds comprises a complex community of microorganisms including bacteria, fungi, archaea and viruses which play synergistic roles to ensure high productivity in the birds [5,6]. Such roles include aiding of digestion and absorption of nutrients, immune system modulation, production of vitamin and amino acids and inhibiting the colonization of potential pathogens [7,8]. An important portion of the bird's gastrointestinal tract (GIT), is the cecum which is known to harbor the highest population of microbes while serving as the major factory for microbial fermentation, thus contributing to the production of volatile fatty acids (VFAs) [9]. Bacteria in the phyla Firmicutes and Bacteroidetes are the dominant colonizers of the caeca, followed by two other phyla, Actinobacteria and Proteobacteria having minor dominance in the caeca [10].

Recent research has shown that feeding BSFLM can beneficially reshape the gut bacterial community in broilers. For example, diets containing BSFL in combination with *Desmodium intortum* was reported to alter the abundance of bacterial phyla, primarily the Firmicutes, Bacteroidetes and Proteobacteria, while the alpha diversity was increased, coupled with boosting the population and activities of beneficial genera such as *Lactobacillaceae* and *Ruminococcaceae* [11,12]. Such observed shift in bacterial population and activity have pronounced effects on gut health, immune modulation, and fermentation patterns. Moreover, bacteria only form a part of the gut microbial ecosystem, there are the methanogenic archaea known for consuming fermentation hydrogen while producing methane as a by-product [13]. However, the key roles played by these specialized organisms are far less well understood in the gut microbiology literature.

Methanogens have been detected in the gastrointestinal tract [14,15]. One of the earliest attempts identified and quantified methanogenic archaea in adult cecum and reported 11 phylotypes, most matching *Methanobrevibacter woesei*, with 16S rRNA gene copy numbers ranging between ∼5.50–7.19 log_10_ copies per gram wet weight in cecal contents [16]. Also, in young broiler chicks aged 3–12 days, methane was detected and the methanogenic archaea in feces were quantified which showed presence of methanogens (order Methanobacteriales) with copy numbers between ∼4.19–5.51 log_10_ per gram wet weight and increases in proportion of positive methane cultures as birds aged [17].

It has been documented that the major archaea players of cecal are *Methanobrevibacter*, *Methanococcus*, *Methanothermobacter*, *Methanosphaera*, *Methanobacterium*, *Methanopyrus* and *Methanothermus* [18]. Furthermore, the methanogenic archaea, *Methanobrevibacter woesei* and other strains have been suspected to play profound roles in chickens’ intestinal fermentation [19,20]. Further, the use of 16S ribosomal RNA gene amplification of chicken caeca has revealed the methanogenic archaea phylum to have a 99 % similarity to *M. woesei* [18]. Most recently, [8], employed metagenome-assembled genomes to reveal new, previously unknown and much higher diversity of archaea in chickens. Despite these findings, information on the poultry methanogenic microbial community is still very limited. Meanwhile, methanogens are important in the GIT as they utilize several end products of bacterial anaerobic fermentation, especially gases (H_2_, CO_2,_ acetic or formic acids) to produce methane, emitted as a by-product of enteric digestion. This invariably helps to maintain hydrogen at low partial pressures and keep the thermodynamic requirements for the anaerobic fermentation processes [21].

Despite these findings, there remains limited insight into the functional methanogenesis pathways (e.g. hydrogenotrophic, methylotrophic, acetoclastic) in broilers, especially under diets that include BSFLM, and how these are modulated in relation to bacterial communities, fermentation products, and host energy efficiency [[22], [23], [24], [25], [26]]. Moreover, while several studies on BSFLM feeding have profiled the bacterial microbiome (with high-throughput sequencing of bacterial 16S rRNA genes), very few address the archaeal community or measure expression of genes central to methanogenesis (e.g. *mcrA*, coenzyme M reductase, hydrogenase genes) or hydrogen flux [[27], [28], [29], [30]]. Also, the competitive or complementary interactions among methanogens and other hydrogen sinks (such as acetogens or sulfate-reducing bacteria) remain underexplored in the BSFLM context. Age and diet inclusion levels likely modulate these dynamics, yet the literature is silent on dose-response or developmental stage effects for methanogenesis in poultry fed insect-based diets. Given the potential environmental implications—methane being a potent greenhouse gas—and the fact that methanogenesis represents fermentative energy that is lost to the host, clarifying how BSFLM influences methanogenic activity is not only of academic interest but potentially of practical relevance [[31], [32], [33]]. If BSFLM can reduce or shift methanogenic activity (without compromising bird performance), this could improve overall energy utilization and reduce emissions in poultry systems.

This study therefore aims to characterize the methanogenic archaeal community in broilers fed diets with varying levels of BSFLM, quantify abundance (and expression, where possible) of key methanogenesis pathway genes, assess shifts in hydrogen-utilizing microbial groups (including methanogens, acetogens, etc.), and relate those microbial shifts to functional outcomes — such as volatile fatty acid profiles, possible methane emission proxies, and energetic or growth performance metrics. We hypothesize that increasing BSFLM inclusion will reduce the abundance or activity of hydrogenotrophic methanogens, increase alternative hydrogen sinks, and improve fermentative energy retention, all while maintaining growth performance.

## 2 Materials and methods

### Ethical approval

Prior to commencement, an ethical approval was sought and obtained from the Bowen University Research Ethical Committee with number BUREC/24/AGR21. The research was also conducted in line with the guidelines approved by the institutional animal care and use committee (IACUC).

### 2.1 Broiler rearing

A total of 50-day-old chicks (DOC) of the *Arbor Acre Plus* were sourced from Farm Supports Hatchery situated at Ile-Ogbo (21 km from Bowen University), Osun State, Nigeria. The birds were assigned to 5 treatments (T_1_-T_5_) using the completely randomized design (CRD), where T_1_ serves as the control (conventional broiler feed). Each treatment contained 15 birds and had 3 replicates with 5 birds in each. They were reared for 8 weeks.

### 2.2 Black soldier fly larvae

A large quantity of BSF larvae or prepupae was obtained from a reliable farm in Iwo, Osun state, to hatch into BSF.

### 2.3 Feeding of BSF

According to Hopkins et al. [34], black soldier flies can address two global challenges, of which organic waste is one. Mohd-Noor et al. [35] stated that wastes, which include municipal waste, wasted food, and industrial food-processing wastes, can be digested by black soldier fly. The larvae of this insect are known to feed on manure produced from livestock wastes [36], as well as plant-based wastes [34]. In this study, organic wastes from poultry, piggery, and cattle were collected from the Bowen University Teaching and Research Farm and were anaerobically co-digested for a period of 30 days hydraulic retention time (HRT) using standard methods [[37], [38], [39], [40], [41], [42]]. The digestate slurry obtained after the completion of digestion was used as feed for the BSF.

### 2.4 Feed-diet formulation

After feeding for a period of 8 weeks, the BSFL was collected, sterilized, and dried, then ground into powder and mixed with other raw materials to formulate five diet types for the broilers. Calculated estimates of the ingredients were used in the formulated diets following the nutrient requirements guidelines for broiler [43]. The BSFL meal was used to replace fish meal (FM) partially and completely at various inclusion levels and was fed to the broilers at the starter and finisher phases, as seen below.

### 2.5 Partial and complete substitution of BSFL meal for fish meal

The partial and complete substitution of BSFL meal for fishmeal is shown in Table 1.

**Table 1:** Partial and complete substitution of BSFL meal for fish meal.

Treatment Code	Diet Description	BSFLM Inclusion (%)	Fishmeal Inclusion (%)	Reference
**T1**	Control diet	0 % BSFLM	100 % FM	
**T2**	Partial substitution	25 % BSFLM	75 % FM	Wahid et al. (2021)
**T3**	Equal substitution	50 % BSFLM	50 % FM	Attivi et al. (2022)
**T4**	High substitution	75 % BSFLM	25 % FM	Nampijja et al. (2023)
**T5**	Full substitution	100 % BSFLM	0 % FM	Purwanti and Nahariah (2020)

### 2.6 Growth performance

The live weight (LW) of birds was recorded individually at their arrival and at the end of each feeding phase. All measurements were performed at a pen level. All weights were taken using electronic scales with an accuracy of 0.1 g (Signum, Sartorius, Bovenden, Germany) [44].

### 2.7 Intestinal sampling

At the end of the experimental trial, birds from each dietary treatment were randomly selected and slaughtered at Bowen University Teaching and Research Farm. Twelve hours before slaughter, the birds had their final meal. At the slaughterhouse, the Broilers were slaughtered humanely. The cecal contents were sampled using a sterilized swab cooled at 40 °C (for a maximum of 2h), collected into sterile plastic tubes, and frozen at 8 °C until DNA extraction.

### 2.8 DNA extraction

Genomic DNA was extracted from the samples following the procedures in the instruction protocol manual of the ZymoBIOMICS DNA Miniprep Kit (Zymo Research, Catalogue No. D4300). Samples were mechanically lysed in bead-beating tubes, the lysate was clarified by centrifugation, and DNA was purified on a silica spin-column using binding and washing buffers supplied with the kit. DNA was eluted in nuclease-free water and stored at −20 °C prior to sequencing [45].

### 2.9 DNA sequencing and 16S bacterial metagenomics workflow

Extracted DNA samples were submitted to a commercial service provider (Inqaba Biotechnogy Industries) for microbiota profiling via full-length 16S rRNA gene sequencing (V1–V9 region). PCR amplification was performed using the universal primers 27F and 1492R (Table 2). The amplicons were barcoded for multiplexing using PacBio M13 barcodes, quantified, pooled in equimolar amounts and purified via AMPure PB beads. A SMRTbell library was subsequently prepared according to manufacturer instructions, and loading, polymerase binding and sequencing were executed on the PacBio Sequel IIe instrument. The workflow followed the SMRT Link software protocol for HiFi (high-fidelity) long-read sequencing in metagenomic applications [46].

**Table 2:** Sequencing primers.

M13 tailed forward primer	/5AmMC6/GTAAAACGACGGCCAGT(N)n
M13 tailed reverse primer	/5AmMC6/CAGGAAACAGCTATGAC(N)n

### 2.10 Bioinformatics and statistical analysis

The 16S rRNA gene sequence data were processed using the QIIME 2 bioinformatics platform (version 2024.2), which provides a reproducible, interactive, and scalable workflow for microbiome data science [47]. Sequence reads underwent quality filtering, denoising and merging through the DADA2 plugin to yield amplicon sequence variants (ASVs). Taxonomic assignment was performed by applying a naïve Bayes classifier trained on the SILVA 138 reference database.

Functional predictions were obtained using the Phylogenetic Investigation of Communities by Reconstruction of Unobserved States 2 (PICRUSt2) pipeline [48]. This approach infers the functional capacity of microbial communities by mapping ASVs to a reference phylogeny, predicting gene family abundances, and then translating them into metabolic pathway predictions. Specifically, PICRUSt2 uses hidden-state prediction to estimate the presence of gene families such as KEGG Orthologs (KOs) for each ASV, based on evolutionary relationships.

The resulting KO identifiers were functionally annotated against the Kyoto Encyclopedia of Genes and Genomes (KEGG) database [49] to identify the associated enzymes and metabolic pathways. Enzyme abundances were aggregated per sample, producing functional profiles expressed as relative abundance values. Only enzymes with non-zero abundance in at least one sample were retained for downstream analysis. Comparative analysis of enzyme profiles across treatments (Control, T2, T3, and T5) was performed to identify differences in predicted metabolic capacities of the gut microbiome. This type of functional metagenomic prediction has been widely applied in poultry microbiome studies to explore the relationship between diet, gut microbial composition, and host physiology [50,51].

### 2.11 Determination of prescence of methanogen

The KEGG functional analysis revealed pathways associated with methanogenesis, indicating the presence of methanogenic microorganisms within the broiler gut.

## 3 Results

### 3.1 Detected pathways of enzymes involving methanogenesis

**Fig. 1:**
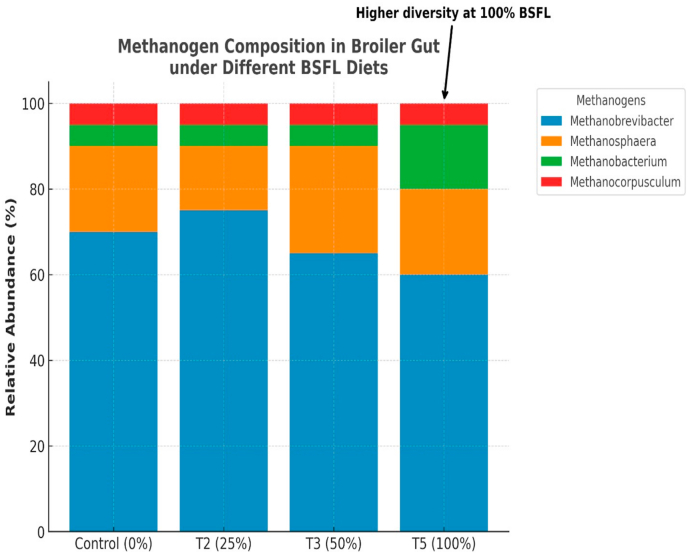
Methanogen Composition of Broiler Gut Under Different BSFL diets.

**Fig. 2:**
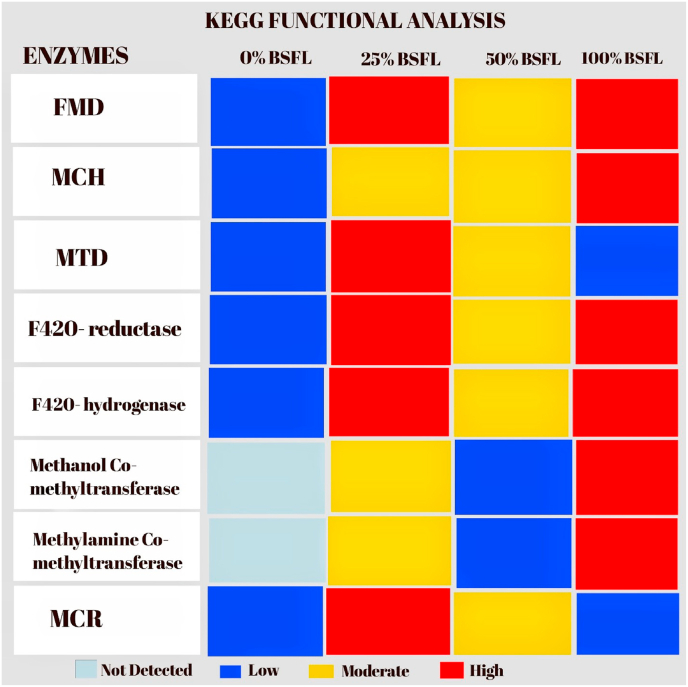
KEGG functional analysis.

Fig. 1 shows all the methanogenic archaeal genera detected in the samples. These includes *methanobrevibacter, methanosoahaera, methanobacterium and methanonocorpusculum*. Analyzed samples from all treatments were found to be positive for methanogenesis. Meanwhile, the number of operational taxonomic units (OTUs) for each sample are 33, 107, 114 and 155 for T1, T2, T3 and T5, respectively which further reveal an increasing progression with increased inclusion of BSFL diet. In terms of relative abundance of the four implicated archaeal genera, the order is *methanobrevibacter* > *methanosoahaera* > *methanobacterium* > *methanonocorpusculum.* Besides, diversity was found to be directly proportional to BSFLM inclusion level with the 100 % BSFLM having the highest diversity. Table 3 shows the detected pathways involving methanogenesis. In the control sample (T1), methanogenesis enzymes were barely active, producing only background methane potential. In sample T2 having 25 % BSFL inclusion, there was the highest detection of enzymes at both hydrogenotrophic (FMD, F420 enzymes) and methylotrophic (methyltransferases) routes which further shows a more pronounced methanogenesis than the control. In sample T3 with 50 % BSFL inclusion, enzymes were present but weaker, possibly due to microbial shifts reducing hydrogen availability while in sample T5 with 100 % BSFL inclusion, there was persistence of methanogenesis enzymes majorly via the methyltransferases, which is also linked to protein-rich BSFL diet which favors methylamine metabolism. The KEGG functional analysis used in the detection of pathways associated with methanogenesis and which indicate the presence of archaea organisms within the broiler gut is further illustrated in Fig. 2. The detected enzymes include Formylmethanofuran Dehydrogenase (FMD), Methenyltetrahydromethanopterin Cyclohydrolase (MCH), Methylenetetrahydromethanopterin Dehydrogenase (MTD), F420-reductase, F420-hydrogenase, Methanol Co-methyltransferase, Methylamine Co-methyltransferase and Methyl-Coenzyme M Reductase (MCR). The control treatment showed low detection of all enzymes except Methanol Co-methyltransferase and Methylamine Co-methyltransferase which were not detected at all. In T2 (25 % BSFL inclusion), FMD, MTD, F420-reductase, F420-hydrogenase and MCR were detected in high proportion while MCH, Methanol Co-methyltransferase and Methylamine Co-methyltransferase were moderately detected. In T3 (50 % BSFL inclusion), all enzymes were moderately detected except Methanol Co-methyltransferase and Methylamine Co-methyltransferase which were detected in low amounts. In the T5 (100 % BSFL inclusion) however, the detection of all enzymes was high except MTD and MCR that were low.

**Table 3:** Detected pathways of enzymes during methagenogenesis.

Enzyme (Name + Korg Orthology)	Function	Control (0 % BSFL)	T2 (25 % BSFL)	T3 (50 % BSFL)	T5 (100 % BSFL)
Formylmethanofuran dehydrogenase (FMD, K00200–K00205)	Initiates CO_2_ reduction → Formyl-MFR	Low; background activity	High; strong hydrogenotrophic methanogenesis	Moderate; reduced activity	Present; low activity
Methenyltetrahydromethanopterin cyclohydrolase (MCH, K01499)	Converts Formyl-H_4_MPT → Methenyl-H_4_MPT	Low detection	Clear presence	Moderate	Weak, reduced
Methylenetetrahydromethanopterin dehydrogenase (MTD, K13942)	Electron transfer in C1 metabolism	Low activity	Strong detection; balanced electron flow	Moderate	Low
Coenzyme F420-dependent methylenetetrahydromethanopterin reductase (K00206)	Redox reaction, reduces methylene-H_4_MPT → methyl-H_4_MPT	Weak presence	Strongly present	Moderate	Reduced
Coenzyme F420 hydrogenase (K03388–K03390)	Provides reducing power (H_2_ → F420H_2_)	Very low	Strong detection; signature methanogen marker	Moderate	Reduced
Methanol-corrinoid protein Co-methyltransferase (K14080–K14083)	Transfers methyl group from methanol	Rare	Moderate presence	Low	Stronger (linked to BSFL proteins)
Methylamine-corrinoid protein Co-methyltransferase (K16188–K16194)	Transfers methyl group from methylamines	Not detected	Detected; moderate	Low	Strong, supports methylotrophic pathway
Methyl-coenzyme M reductase (MCR, K00399, K00401, K00402)	Terminal enzyme → methane formation	Low; background methanogenesis	Strongest activity	Present, reduced	Present but weaker

## 4 Discussion

Although methanogens are less prevalent in poultry than in ruminants, their metabolic pathways still appear in functional annotations, underscoring their role in gut ecology [52]. The key Enzymes identified include Methyl-Coenzyme M Reductase (MCR), KO: K00399, K00401, K00402 which is a defining feature of all methanogens. It facilitates the final reaction in methanogenesis i.e., the reduction of methyl-coenzyme M (methyl-CoM) with coenzyme B to yield methane and a heterodisulfide. Besides, the presence of MCR genes strongly indicates methanogenic activity, as no other microorganisms outside of methanogens possess this enzyme. Another important enzyme is the Formylmethanofuran Dehydrogenase (FMD); KO: K00200 – K00205 known for catalyzing the initial step for the reduction of CO_2_ into formylmethanofuran. This crucial step helps to integrate CO_2_ into the methanogenesis pathway, linking microbial CO_2_ utilization with methane production. This also suggests a potential for hydrogenotrophic methanogenesis (CO_2_ + H_2_ → CH_4_). Methenyltetrahydromethanopterin Cyclohydrolase (MCH); KO: K01499 is another enzyme detected in this study and it is known for transforming methenyl-H_4_MPT into formyl-H_4_MPT, a key step in the H_4_MPT-dependent C1 carrier pathway. Its presence implies an active functional C1 transfer pathway functioning within the gut methanogens. Methylenetetrahydromethanopterin Dehydrogenase (MTD); KO: K13942 was also detected as an important enzyme in methanogenesis. This enzyme facilitates the reduction of methenyl-H_4_MPT, further directing electrons towards the production of methane. The presence of this enzyme in the study affirms the occurrence of stepwise reduction of C1 units. Another enzyme cocktail detected in the study is the Coenzyme F420-dependent Enzymes, F420-dependent methylenetetrahydromethanopterin reductase (K00206), F420 hydrogenase (K03388–K03390). These enzymes utilize coenzyme F420, which is a distinct cofactor predominantly found in methanogens. They are known for providing the necessary reducing equivalents (H_2_/CO_2_ metabolism) to the pathway and their identification supports genuine methanogenic activity and often serves as a genetic marker for archaeal methanogens. The last enzymes cocktail detected in the study is the Methyltransferases (Methanol and Methylamine Pathways); KO: K14080–K14083 (methanol-corrinoid protein Co-methyltransferase); KO: K16188–K16194 (methylamine-corrinoid protein Co-methyltransferase). These enzymes facilitate the transfer of methyl groups from methanol or methylamines to coenzyme M. Their presence further indicates that methylotrophic methanogenesis (utilizing methanol or methylamines) is taking place in the broiler's gut microbiota, revealing diversity in the methanogen metabolic strategies that extend beyond simple CO_2_ reduction.

Methanogens utilize hydrogen generated by fermentative bacteria (e.g., Firmicutes, Bacteroidota), mitigating the accumulation of hydrogen that could hinder the efficiency of fermentation. As found in this study, the presence of methanogens sustained a balanced redox state in the gut, by indirectly supporting enhanced production of short-chain fatty acids (SCFAs), which aids in the host's energy metabolism [31]. This research exhibited methanogenesis pathways across various treatments, although their relative abundance fluctuated with lower BSFL inclusion (T2), a more harmonized gut ecosystem with moderate methanogen activity was observed. At higher inclusion (T5), altered microbial diversity may influence hydrogen availability, which could affect methanogen activity and this agrees with the report of Mahayri et al. [30]. The presence of MCR and F420-dependent enzymes serves as definitive proof of methanogenic activity. The pathways identified indicate that both hydrogenotrophic (CO_2_-reducing) and methylotrophic routes are operational. Methanogens are a smaller yet functional part of the broiler gut microbiome. The study therefore affirms that inclusion of BSFL alters microbial ecology but does not eliminate methanogenic potential which is in alignment with a previous submission [23].

The identification of significant methanogenic enzymes in this research suggests that methanogenic archaea actively contribute to the gut microbiota of broilers. Their presence, quantity, and pathway utilization seem to vary based on the proportion of black soldier fly larvae (BSFL) incorporated into the diet. In the control sample (0 % BSFL), methanogenic enzymes were detected only at minimal levels, indicating the existence of low-abundance hydrogenotrophic methanogens like *Methanobrevibacter* and *Methanobacterium*. This observation aligns with the general understanding that poultry maintain lower levels of methanogens compared to ruminants, where hydrogenotrophic methanogenesis is predominant [53]. At 25 % BSFL inclusion (T2), methanogenic activity was at its highest, with significant detection of formylmethanofuran dehydrogenase, methylenetetrahydromethanopterin dehydrogenase, and coenzyme F420 hydrogenase. This enzymatic pattern strongly indicates the dominance of hydrogenotrophic methanogens, particularly *Methanobrevibacter*, *Methanoculleus*, and *Methanospirillum*. These groups are well recognized for their ability to utilize hydrogen and carbon dioxide to generate methane, thereby enhancing fermentation by mitigating hydrogen accumulation [54]. The prevalence of hydrogenotrophic pathways at this level of dietary inclusion suggests that moderate amounts of BSFL support fermentation efficiency by ensuring appropriate hydrogen turnover.

Conversely, at 50 % BSFL inclusion (T3), there was a noticeable transition toward mixed methanogenic pathways, with intermediate activity observed in both hydrogenotrophic and methylotrophic enzymes. This indicates a transitional community phase where *Methanobrevibacter* is present alongside versatile methanogens such as *Methanosarcina*. Importantly, *Methanosarcina* can utilize both CO_2_/H_2_ and methyl compounds, making it well-suited for adapting to fluctuations in substrate availability. This transitional state aligns with findings that diets containing BSFL modify poultry gut microbiota, enhancing diversity and changing functional capabilities [11].

At 100 % BSFL inclusion (T5), methanogenic activity significantly shifted towards the methylotrophic pathway, as evidenced by strong detection of methanol and methylamine corrinoid protein Co-methyltransferases. Likely dominant genera include *Methanosarcina*, *Methanomethylophilus*, and *Methanomassiliicoccus*, known for utilizing protein-derived methylamines and methanol. These findings are in line with research indicating that high-protein diets supply ample substrates for methylotrophic methanogenesis [32]. In poultry that consume BSFL, shifts in the microbial community towards taxa adapted to protein-rich substrates have been recorded (Siw et al.*,* 2022). The reliance on methylotrophic methanogens in a full BSFL diet implies changes in nitrogen turnover and potential effects on gut pH due to protein fermentation byproducts.

These changes have significant functional implications. Hydrogenotrophic methanogenesis observed in T2 improves fermentation efficiency by managing hydrogen partial pressure, while methylotrophic methanogenesis noted in T5 may reflect adaptation to high-protein diets, impacting nitrogen cycling and gut metabolite equilibrium. Such changes in methanogen ecology are consistent with wider evidence indicating that BSFL-based diets alter gut microbiota in poultry and pigs, promoting microbial richness and modifying metabolic pathways [4,11,12]. In all, the major methanogenic genera implicated in this study aligns with those already reported as colonizers of the caeca of poultry birds [14,15,18]. The dominance of *methanobrevibacter* also agree with earlier report that it is usually the most abundant general in poultry caeca samples [20].

## 5 Conclusion

This study has stood out, to the best of our knowledge, as the first to evaluate the effect of BSFLM dietary inclusion on the caeca methanogenic signature of broilers. The various inclusion level of the BSFLM showed varied impact on the caeca archaeal composition, causing changes in the relative abundance of all detected methanogens with the pronounced effect at 100 % BSFLM inclusion level. Enzymatic activities also varied from minimal (0 % BSFLM inclusion) to strong detection of hydrogenotrophic pathway enzymes and moderate presence of methylotrophic methyltransferases, suggesting dominance of hydrogenotrophic methanogenesis in the 25 % inclusion level. This moved to moderate hydrogenotrophic enzyme activity, with weaker methylotrophic components with 50 % BSFLM inclusion and to highly pronounced methylotrophic pathway enzymes, while hydrogenotrophic enzymes persisted but at low levels with 100 % BSFLM inclusion. These shifts have implications for gut fermentation efficiency, substrate availability, nitrogen metabolism, and possibly GHG emission. Optimization of BSFLM inclusion levels may help balance production performance and environmental sustainability. Future research should incorporate direct measurements of methane emissions in response to BSFLM intake in order to obtain valuable insights into the relationship between methanogen diversity and methane production. These will contribute to the development of sustainable poultry production, with environmental protection.

## Declaration of generative AI and AI-assisted technologies in the writing process

During the preparation of this work, the authors used no generative AI and AI-assisted technologies. The authors reviewed and edited the content as needed and take full responsibility for the content of the publication.

## Funding

This research received no funding whatsoever.

## CRediT authorship contribution statement

**Deborah Oluwaferanmi Ibiwoye:** Investigation, Methodology, Resources, Software, Validation, Writing – original draft, Writing – review & editing. **Opeyemi Adetola Oladejo:** Investigation, Methodology, Supervision, Visualization, Writing – original draft, Writing – review & editing. **Oluwatomisin Aderonke Akinsola:** Formal analysis, Investigation, Software, Writing – original draft. **Aruna Olasekan Adekiya:** Formal analysis, Investigation, Resources. **Olufemi Mobolaji Alabi:** Formal analysis, Resources, Validation. **Ayantade Dayo Ayansina:** Methodology, Resources, Supervision. **Samuel Olatunde Dahunsi:** Conceptualization, Funding acquisition, Investigation, Methodology, Project administration, Resources, Supervision, Writing – original draft, Writing – review & editing.

## Declaration of competing interest

The authors declare that they have no known competing financial interests or personal relationships that could have appeared to influence the work reported in this paper.

## Data Availability

No data was used for the research described in the article.
